# Genetic and Epigenetic Approaches to Opioid Use Disorder

**DOI:** 10.1017/erm.2025.10024

**Published:** 2025-09-25

**Authors:** Nadeeka Dimuthu Ranadeva, Praba Jalini Wijekumar, Caroline Anastasia Fernando, Akila Randika Jayamaha, Nafeesa Noordeen, Sureka Chackrewarthy, Neluka Fernando

**Affiliations:** 1 Department of Biomedical Science, Faculty of Health Sciences, KIU, Sri Lanka; 2 School of Life Sciences, Faculty of Science and Engineering, Anglia Ruskin University, Cambridge, UK; 3 Research and Innovation Division, KIU, Sri Lanka; 4 Faculty of Graduate Studies, University of Sri Jayewardenepura, Nugegoda, Sri Lanka; 5 Faculty of Medicine, University of Colombo, Sri Lanka; 6 Faculty of Medicine, University of Kelaniya, Sri Lanka; 7 Faculty of Medical Sciences, University of Sri Jayewardenepura, Sri Lanka

**Keywords:** DRD2, epigenetics, GAD1, heroin, OPRM1, opioid addiction, opioid use disorder

## Abstract

**Background:**

Opioid use disorder (OUD) is a major global-scale social issue affecting public health. The high potential for addiction and dependence makes opioid use a significant concern, contributing to substance-related disorders. Both genetic and environmental factors contribute to the predisposition to OUD, with the opioidergic, dopaminergic, and GABAergic systems playing primary roles in itsonset.

**Methods:**

This narrative review documents the association between genes and their variants related to these three systems, along with current evidence on epigenetic interventions in OUD. Relevant studies investigating candidate-gene associations and molecular mechanisms were synthesized to highlight genetic variants and epigenetic processes linked to OUD.

**Results:**

Genetic associations play a prominent role in OUD, with several single-nucleotide variants identified in affected populations. Key genes implicated include OPRM1, OPRD1, OPRK1, PDYN, OPRL1, and POMC from the opioidergic system; DRD1, DRD2, DRD3, DRD4, ANKK1, and COMT from the dopaminergic system; and GABRA2, GABRB3, GABRG2, GAD1, and GAD2 from the GABAergic system. Evidence also indicates that chronic opioid use is associated with epigenetic changes through posttranslational histone modifications and DNA methylation. However, limitations in existing studies include small sample sizes, limited replication, and potential stratification biases.

**Conclusions:**

Although many candidate-gene associations have been proposed for OUD, robust evidence remains limited. Large, ancestrally diverse genome-wide association studies (GWAS) and systematic replication studies are urgently needed. A deeper understanding of the genetic, epigenetic, and neurobiological bases of addiction will be essential for the development of precisely targeted medications to improve prevention and treatment outcomes for OUD.

## Introduction

Substance use disorders (SUDs) are multistage conditions (which typically progress through various stages, from initial substance use to dependence and addiction) characterised by exposure to addictive substances that result in dependence and addiction. Opioids are among the most commonly misused substances, known for their elevated risk of causing addiction and dependence (Ref. 1). Opioid tolerance, dependence and addiction are all caused by chronic opioid abuse, which can lead to opioid use disorder (OUD). Clinically, OUD is a persistent, relapsing disorder marked by physical and psychological symptoms that promote opioid-seeking behaviour. The 2013 Diagnostic and Statistical Manual of Mental Disorders of the American Psychiatric Association defines OUD as the recurrence of two or more of 11 problems within 12 months, such as withdrawal symptoms, opioid seeking and excessive time spent using opioids (Ref. 2). OUD is prevalent among diverse ethnic and age groups.

### Global trends in opioid usage

The World Drug Report 2023 (Ref. 3) reported that 296 million individuals around the world used drugs in 2021 (Ref. 3). This is 5.8% of the population aged 15–64 and a 23% rise over the preceding 10 years. According to the United Nations Office on Drugs and Crime, 61 million people around the world take opioids for non-medical reasons (Ref. 3). This was a big jump from the 26–36 million people who did so in 2010. Opioids are still the most dangerous substance. In 2019, they were responsible for approximately 70% of the 600,000 drug-related deaths, including about 150,000 fatal overdoses (Ref. 4).

### Age-group prevalence and regional patterns

It is observed that drug use has an uneven distribution across the world. According to reported data, it has been indicated that in 2021, 39.5 million people were victims of a drug use disorder (Ref. 3). The contrasting regional patterns are also noteworthy: the use of opioids is known to be increased in Africa, Asia, Europe and North America, where cannabis remains the most widely used substance in North and South America and Asia (Ref. 3). Age-specific prevalence was also noted, where the majority were in the young adult category, aged 18–35 years. Peer influence and living environment were identified as the main risk factors (Ref. 3). In the South Asian region, the prevalence of opiate use has been reported as 1.1% of the adult population. This accounts for nearly twice the global average (Ref. 5). Synthetic opioids such as fentanyl can be found in North America predominantly, often creating an overdose crisis, while tramadol leads the misuse in the West African region (Ref. 3).

### Treatment gaps and global access challenges

Although there is an increasing prevalence of OUD, access to treatment remains limited. Less than 9% of individuals who were victims of drug use disorders in 2021 had received any form of treatment, which can be pointed out as a drop from 11% reported in 2015 (Ref. 6). The lowest treatment rates were reported in Africa (2.8%) and Asia (5.1%), whereas in the Americas, it has been reported as 10.7% (Ref. 6). Women have been found to represent 25% of drug use disorders; however, it has been noted that women remain undertreated for the condition, accounting for only about 20% of those receiving treatment (Ref. 6). Access to essential opioid medications for pain and palliative care also has an unequal distribution. About 87% of the world population has no adequate access to the necessary medications, emphasizing a 46-fold disparity between high- and low-income countries (Ref. 3). These gaps highlight the urgent need for evidence-based, equitable, and gender-sensitive treatment expansion.

### The multifactorial nature of drug addiction

There are three aspects to the nature of drug addiction: biological, psychological and social (Ref. 7). Individual risk factors for developing a SUD (including OUD) can include genetic predisposition as a biological factor, family background and peer influence as social factors, and psychiatric conditions, as well as personality traits as psychological factors (Ref. 8). The development of OUD can be attributed to both genetics and environment. For example, individuals who are predisposed to addiction have significantly worse outcomes when they reside in environments with easy access to intoxicating substances and few restrictions on their use. Among these risks, genetics is considered a major contributory factor for addiction development. There is a 40–60% genetic contribution to developing an addiction to drugs (Refs 9, 10, 11). It has been demonstrated that genetic factors influence the susceptibility to developing addiction in general, where specific genetic variants can make individuals vulnerable to particular substances or types of addiction (Ref. 1).

This narrative review has been written with the aim of capturing the knowledge regarding the genetic and epigenetic determinants of OUD. Emphasis is given to the findings related to the opioidergic, dopaminergic and GABAergic neurotransmitter systems. Further, we examine known gene variants and epigenetic modifications linked to OUD. A rigorous assessment of the robustness of evidence for these associations across various populations has also been indicated. Epigenetic aspects related to OUD and genome-wide association studies (GWAS) evidence related to OUD have also been indicated. The existence of a knowledge gap in the field has been indicated while suggesting areas for future research. The primary aim is to elucidate how genetic and epigenetic research can enhance the comprehension of OUD aetiology and inform tailored treatment approaches for this condition.

## Opioid use disorder mechanism

Opioids are natural or synthetic chemicals that reduce pain by interacting with opioid receptors on the cell surface. Opioids include the illicit drug heroin, lab-made opioids such as fentanyl and legally available painkillers such as morphine, oxycodone, hydrocodone and codeine (Ref. 12).

Opioids exert their effect by travelling through the blood to the brain and binding to opioid receptors on the cell surfaces of opiate-sensitive neurons (Ref. 13). There are three types of opioid receptors: the mu opioid receptor (MOR), the delta opioid receptor (DOR) and the kappa opioid receptor (KOR). Enkephalin and β-endorphin are the two main endogenous ligands for the MOR. Dynorphins and enkephalins preferentially bind to KOR and DOR, respectively (Refs 14, 15). When opiates bind to receptors, the brain’s reward system is activated, resulting in feelings of pleasure while engaging in brain-promoting activities such as eating (Ref. 16).

Opioids (morphine, codeine, opium) are frequently prescribed for the treatment of moderate to severe pain, which controls pain, relieves cough and diarrhoea and, due to prolonged use, also induces euphoria and stimulates the brain’s reward system (Ref. 17). The patient continues to use opioids despite the development of serious health issues, which results in OUD. A patient with drug addiction, which is a type of SUD, may become dependent on higher doses of drugs to achieve the desired feeling of pleasure, which can result in fatal conditions such as respiratory distress.

**Figure 1.**
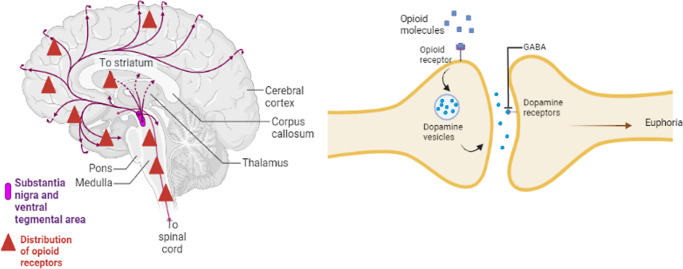
Distribution of dopamine (Dopaminergic system) - Left, opioid receptors, GABA, and mechanism of OUD in the Human Brain – Right. The diagram represents the key pathways of the brain involved in development of Euphoria induced by opioids and the development of OUD. **Left:** Dopaminergic neurons are represented in blue in the ventral tegmental area (VTA) and projects to the nucleus accumbens (NAc) in the mesolimbic reward pathway. **Right:** Opioids (e.g., heroin, morphine) bind to the mu-opioid receptors (MOR, depicted in red) on GABAergic interneurons in the VTA. This binding inhibits the release of GABA because the activaton of MOR causes the hyperpolarization of GABA neurons. **GABA** (depicted in green) is an inhibitory neurotransmitter that normally suppresses the firing of VTA dopamine neurons. By inhibiting GABAergic neurons, opioids **disinhibit** the dopaminergic cells The outcome is an enhanced release of dopamine in the nucleus accumbens (NAc) and the prefrontal cortex. The rise in dopamine levels (represented in blue) within these areas leads to the strong sensations of pleasure or euphoria linked to opioid consumption, reinforcing the behavior of taking drugs.To sum up, the illustration indicates that opioids promote increased dopamine release by inhibiting GABA’s inhibitory action – an essential process in the reward pathway. Important brain regions (VTA, NAc, prefrontal cortex) and neurotransmitters are identified in the diagram. (Note: To enhance clarity, the anatomical labels in the original figure have been magnified.)

Opioids simply stimulate the mesolimbic (midbrain) reward system. This system generates signals in the ventral tegmental area (VTA) that cause the nucleus accumbens to release the neurotransmitter dopamine. This dopamine stimulation underlies the effect on pleasure sensations. Opioids prevent dopamine cells from inhibiting dopamine release. This allows dopamine to enter the nucleus accumbens and prefrontal cortex of the forebrain, which plays a crucial role in drug dependence (Ref. 13). Moreover, drug addiction has been linked to a decrease in GABA communication between the VTA and the nucleus accumbens (Ref. 18). GABA is an inhibitory amino acid neurotransmitter that modulates the release of dopamine, serotonin, noradrenaline and glutamate, which can affect neurotransmitter systems (Ref. 19). These medications can cause the striatum to release more dopamine and glutamate while decreasing GABA production in the thalamus (Ref. 20). Other parts of the brain create a long-term memory that links these positive emotions to their circumstances and surroundings. This frequently results in a drug craving (Ref. 13) (Figure 1).

## Genetics and OUD

Many studies have examined the genetic causes of SUDs and have investigated the genes associated with OUD. However, over the past decade, the studies have concentrated on the single-nucleotide variants of genes reported to be associated with OUD. Numerous groups have hypothesised that addictions are complex disorders that may be influenced by the polymorphisms of many genes. Certain studies have also investigated whether a general set of genetic variables increases or decreases susceptibility to addiction (Ref. 21).

There are diverse types of evidence to establish the genetic link between drug dependence and its aetiology. Family, adoption and twin studies were carried out in the early phase to examine substance dependence and abuse, supporting the theory that there is a substantial genetic component to drug addiction (Refs 10, 22). Further studies were carried out as candidate gene studies and GWAS (Ref. 23). The earliest studies used to establish a genetic contribution to specific addictions focused on the genetic epidemiology of alcoholism (Ref. 23). Recent epidemiological research has revealed both similarities and differences in the heritability of other addictions, especially OUD (Ref. 24). On the basis of twin and family studies, heritability estimates for OUD risk range from 23% to 54% (Ref. 10, 22).

Addiction, particularly opioid addiction, is influenced by specific genes and single-nucleotide variants. Knowledge of this aspect of the study will improve understanding of the mechanism of drug addiction and the identification of vulnerable individuals.

## Opioidergic system

There are three types of opioid receptors: mu-opioid receptor (MOR), kappa-opioid receptor (KOR) and delta-opioid receptor (DOR). The MOR is encoded by the OPRM1 gene, which has been intensively studied in heroin addiction and other opioid addictions, as well as in methadone and buprenorphine-based treatments for drug addiction. The OPRM1 variant rs1799971, which results in the Asn40Asp amino acid substitution of the MOR (Ref. 25), is one of the most extensively researched variants in the field of addiction (Ref. 26). The rs1799971 variant significantly reduces OPRM1 mRNA expression, which leads to low levels of MOR and high binding affinity of the endogenous ligand, β-endorphin (Refs 27, 28).

The KOR systems (encoded by OPRK1) and their endogenous KOR agonist ligands, the dynorphins (encoded by PDYN), modulate addictive states, such as addiction to short-acting MOR agonists (Ref. 15). The DOR is encoded by the OPRD1 gene. Although the common opioid drugs that cause dependence do not primarily target the DOR, the receptor is involved in reward pathways of the neurobiology of drug addiction (Ref. 29).

## GABA-ergic system

GABA (gamma-aminobutyric acid) is the principal inhibitory neurotransmitter in the brain. It acts through the ionotropic GABA-A and metabotropic GABA-B receptors (Ref. 30). The GABA-A receptor belongs to the ligand-gated ion channel receptor superfamily, with α1–6, β1–3, γ1–3, δ, ε, θ, π and ρ1–3 subunits. GABA-A receptors have primarily been linked to alcohol abuse (Ref. 31). As there is a correlation between GABA-A receptor genes and the mechanism of OUD development, it is hypothesised that several GABA-A receptor genes may affect the susceptibility of individuals to drug (opioids) dependence. GABRA1, GABRA2, GABRA4 and GABRB1 are genes on chromosome 4p13–12 that encode GABA-A receptor subunits (Ref. 32).

Preclinical research suggests that GABRA2 may play a role in drug addiction (Refs 33, 34). It is interesting to note that Enoch et al. (Ref. 35) assert that GABRA2 variants can predict addiction vulnerability, specifically for heroin addiction, and that childhood trauma and GABRA2 variants can influence addiction risk, specifically for cocaine dependence.

## Dopaminergic system

D1, D2, D3, D4 and D5 are the five subtypes of dopamine receptors. The D1 and D5 dopamine receptors belong to the D1-like family of dopamine receptors, while the D2, D3 and D4 receptors belong to the D2-like family. D1-like family receptor activation is coupled to the G protein, which then activates adenylyl cyclase, whereas D2-like family receptor activation inhibits cAMP formation by inhibiting adenylyl cyclase. The rewarding effects of opioids are mediated by dopamine release and post-synaptic receptor activation. DRD2 is a gene that encodes the dopamine D2 receptor located near ANKK1, a gene that encodes a serine/threonine protein kinase. The DRD2/ANKK1 locus contains two widely studied polymorphisms: rs1800497, a missense variant in ANKK1 exon eight and rs1079597, located in DRD2 intron one. Rs1800497 and rs1079597 are often inherited together rather than by chance in non-African populations (Ref. 36). Both variants are reported among the Han Chinese population with opioid dependence (Ref. 37).

Table 1 provides a brief overview of the genes and the variants studied among the varying ethnicities and their association with OUD. As highlighted in Table 1, various variants within the opioidergic system exhibit associations with OUD that are specific to certain populations. For example, the extensively researched OPRM1 variant rs1799971 (A118G) is more prevalent in non-African populations (Refs 26, 39, 40, 41) and has been associated with the risk of OUD across different cohorts, whereas rs1799972 shows greater effects in individuals of African descent (Refs 26, 38). Additionally, OPRD1 variants such as rs2236861 and rs2236857 have been consistently linked to heroin dependence among European and Israeli populations (Refs 15, 43, 44).

**Table 1.** Genes and SNVs of the reward system of OUD which have demonstrated an association

System	Gene	Function	SNVs	Association	References
Opioidergic	OPRMI	Receptor	rs1799972	Predominantly associated with individuals of African descent (*p* < 0.006) and observed increased frequency of opioid addiction among multiple groups of African American, Caucasian, Hispanic, Native North American or Other ethnicities	[26,38]
			rs1799971	Well-studied variant among varying ethnic groups and more common in non-African populations	[26,39–41]
			rs3778150	Significantly associated with opioid dependence in African-Americans and European-Americans and with heroin dependence among multiple groups; European American, European Australian and African American participants (*p* = 4.3e–8)	[38]
	OPRKI	Receptor	G36T	Two haplotypes (AGCTCGTC, GGCGTGCC) are significantly associated with opioid addiction in Hispanics. The variant is slightly higher in opioid-dependent Caucasians and Hispanics	[38]
	PDYN	Ligand	rs1022563	Significantly associated with opioid addiction in European-Americans	[42]
			rs910080	Significant associations (ORs) for rs910080 and rs199774 were found in female opioid addicts as well as European-Americans (*p* < 0.05)	[42]
			rs199774		
	OPRD1	Receptor	rs2236861	Highly associated with opioid abuse or dependence risk among the European population (*p* = 0.001) and heroin dependence among multiple groups, US Caucasians and Israeli Jewish cases with US Caucasian controls (*p* = 0.002)	[43,44]
			rs2236857	Significantly associated with opioid dependence	[15]
			rs3766951	Significantly associated with opioid dependence	[15]
			rs2234918	The rs2234918 variant is rarely associated with dependence in the German and European populations (*p* < 0.001)	[45,46]
			rs1042114	rs1042114 is associated with opioid dependence in European-Americans. The GCAACT haplotype, which consists of both rs1042114 and rs2234918, is more prevalent in cases of opioid dependence than in controls	[45]
	OPRL1	Receptor	rs6090041	Significantly associated with European population (*p* = 0.03) for opioid exposure and opiate addiction among the Scandinavian population	[47]
			rs6090043	Significantly associated pointwise with opiate addiction among the Scandinavian population	[47]
	POMC	Protein	rs934778	Specifically associated with opiate dependence and associated with minor allele frequencies of 0.30 and 0.27	[48]
			rs10009388		
GABA-ergic	GABRA2	Receptor	rs11503014	Associated heroin addiction in African American (*p* = 0.001)	[49]
	GABRB3	Receptor	rs4906902, rs8179184, rs20317	Contribute to the pathogenesis of heroin dependence	[49]
			rs7165224	Associated with heroin addiction among the African American population (*p* = 0.01)	[50]
	GABRG2	Receptor	rs211014	Associated with heroin dependence in an American (*p* = 0.0005) and Chinese male population (*p* = 0.015)	[51,52]
	GAD1	Protein (Glutamic acid decarboxylase1)	rs1978340, rs3762556, rs3791878, rs3749034, rs2241165	Associated with the development of heroin dependence was reported in the Han Chinese sample	[53]
			rs2058725	Associated with heroin addiction among African Americans (*p* = 0.0074)	[50]
	GAD2	Protein (Glutamic acid decarboxylase 2)	rs8190646	Associated with heroin addiction among African Americans (*p* = 0.0066)	[50]
Dopaminergic	COMT	Enzyme	rs4680	Significant association of the G/A and A/A genotypes with opiate addiction in women, but not in men, among Hispanic subjects with opiate dependence. This result confirmed a previous association report for this variant in an Israeli population (*p* < 0.05). Associated with opioid addiction among European population (*p* = 0.0028)	[54,55]
	DRD1	Receptor	rs5326	Associated with heroin addiction in African Americans and there is an association of rapidity of the heroin dependence with the first dose use among Han Chinese population	[50,56]
			rs686	Associated mixed population of Caucasians and African Americans (*p* < 0.05)	[56]
	DRD2	Receptor	rs1079597	rs1800497 and rs1079597 are inherited together more often. Both variants have been associated with opioid dependence in Han Chinese Associated with European and Chinese (*p* < 0.05)	[57–61]
			rs1800497	Associated with opioid addiction and it is a risk factor for OUD among Chinese and European (*p* < 0.05)	[61–63]
	DRD3	Receptor		Associated with opioid addiction in terms of increased drug seeking which is a risk factor	[64]
	DRD4	Receptor		The 7-repeat variants of DRD4 exon III long repeat alleles are more prevalent in opioid-dependent populations	[64]
			rs1800955	Found to be nominally associated with opioids among Hungarian patients	[61]
	ANKK1	Protein	rs1800497	rs1800497 and rs1079597 are inherited together more often. Both variants have been associated with opioid dependence in Han Chinese	[57,58]

Variants in the GABAergic pathway also display risk signals that are specific to ethnicity: GABRA2 rs11503014 has been linked to heroin addiction in African American individuals, while polymorphisms in GAD1 show significant associations within Han Chinese groups. Furthermore, GABRG2 rs211014 has been independently associated with OUD in both American and Chinese male populations, indicating shared biological mechanisms across different ancestries.

In the dopaminergic system, significant variants include DRD2/ANKK1 rs1800497 (Taq1A) and DRD2 rs1079597, both of which are related to OUD risk in European and Han Chinese populations. Other polymorphisms, like COMT rs4680 (Val158Met), demonstrate associations that vary by sex, particularly being linked to opiate addiction in Hispanic women.

Collectively, these findings highlight that while numerous risk loci are involved in different neurotransmitter systems, the robustness and reproducibility of these associations tend to differ based on ancestry, sex or environmental factors. This reinforces the necessity for replication efforts that are ancestrally diverse and adequately powered to authenticate candidate variants identified in smaller studies.

## Epigenetics and OUD

Previous research has demonstrated a genetic link to OUD. The genetic role refers specifically to the association between OUD and particular genes, including their single-nucleotide variants. The other component of OUD causation is the influence of the individual’s environment. In this context, the interaction of genetics and environment can be described through epigenetic processes. The risk of drug abuse is associated with epigenetic mechanisms that explain some of the interactions between genes and the environment (Ref. 65). Therefore, epigenetics is the study of DNA-independent alterations in the regulation of gene activity and expression. These modifications can occasionally be inherited (Ref. 66).

Chronic substance use (repeated exposure) is partially linked to neuro-adaptive epigenetic changes in people’s brains that result in SUDs (Refs 67, 68, 69). DNA methylation, chromatin remodeling, non-coding RNA and histone modifications all contribute to epigenetic events (Ref. 68). Even though all cells in the human body contain the same genetic information, epigenetic regulatory systems permit the development of several types of cells that, in response to the environment, form skin, heart, kidney, liver and nerve cells. These epigenetic marks can affect the health of offspring and even the expression of inherited traits. When a person uses heroin, for example, the drug can alter the DNA, increasing the production of addiction-related proteins. There is a correlation between elevated levels of these altered proteins and drug-seeking behaviour. There are studies widely carried out *in vivo* animal studies investigating these. Histones are similar to protein threads in that they provide an organisational framework for genes. The loosening or tightening of DNA around histones, exposing or concealing genes, in turn regulates gene expression. Drug exposure can modify gene expression in localised brain regions by affecting specific histones (Ref. 70). According to scientific evidence, manipulating enzymes that alter histones and proteins that bind to them may be an effective method for treating SUDs (Refs 71, 72).

Changes in DNA methylation have been observed after chronic exposure to multiple rewarding drugs, including opioids (Refs 73, 74, 75, 76). In particular, Hwang et al. (Ref. 76) found that increased DNA methylation of the promoter regions of the opioid receptor gene resulted in downregulation of the gene. It is consistent with previous research that MeCP2 can suppress the expression of the MOR gene (Refs 73, 74). Although evidence is limited, it has been suggested that the levels of DNA methylation vary between individuals of different racial groups who are exposed to opioids (Ref. 74). Opioid exposure is known to be linked to germline changes that can be carried down to future generations via transgenerational epigenetic inheritance (Refs 77, 78, 79). Transgenerational epigenetic inheritance refers to phenotypic variation that does not result from variations in DNA base sequences but is passed down through the germline to the progeny, even when no direct opioid exposure occurs. More extensive genetic and epigenetic studies will be required to compare individual and group racial differences in OUD susceptibility or resistance (Ref. 80). The previous studies have shown that genetic and epigenetic markers vary between ethnic groups (Ref. 81). Thus, analytical studies should focus more on determining the contribution of different epigenetic mechanisms to OUD development, which can be used for personalised treatments.

### Recent advances epigenetics and OUD

#### DNA methylation in OUD

It has been found in recent studies that there are consistent DNA methylation changes that may be connected to OUD. One of the main focuses in these studies is the OPRM1 gene that encodes the mu-opioid receptor. Elevated levels of methylation of the promoter region of the OPRM1 have been discovered to decrease receptor expression and may have a connection in turn to tolerance and dependence (Ref. 82). On the contrary, Agulló et al. (Ref. 83) reported a different finding from this pattern. This study was reported to have been done using 345 chronic pain patients who were on long-term opioid therapy. Out of these individuals, those who had OUD showed significantly lower methylation levels of OPRM1 compared to individuals without OUD. The hypomethylation of OPRM1 was also linked to worse pain relief, increased depressive symptoms and comparatively increased rates of adverse events related to opioid use, with significant sex-specific differences (Ref. 83)

Wide-scale epigenomic studies have evidence to indicate that OUD is linked to widespread dysregulation of DNA methylation. A research study by Rompala et al. (Ref. 84) examined the neuronal methylomes and hydroxymethylomes in postmortem tissue from the orbitofrontal cortex, identifying more than 1,700 differentially hydroxymethylated CpG sites in cases of OUD. These sites were related to genes in Wnt pathways, GPCR signaling and neurogenesis, while many loci overlapped with risk loci of psychiatric disorders. Some genes that showed epigenetic alterations were also pharmacological targets of opioid treatments. This may prove the direct link of epigenetic changes to potential therapeutic pathways (Ref. 84).

Perinatal studies further support the clinical significance of methylation changes. One study showed that lower levels of placental methylation of the transporter gene ABCB1 were linked with more serious neonatal opioid withdrawal syndrome. Also, it was discovered that higher CYP19A1 methylation is associated with increased opioid metabolites in umbilical cord blood. The study results of these studies suggest that looking into placental epigenetics as a useful approach may serve to identify biomarkers for neonatal risk (Ref. 85).

#### Histone modifications and chromatin in OUD

The other main epigenetic factor, histone modifications, has also been implicated in OUD occurrence. A study conducted as a case control study (51 OUD cases, 51 controls) by Corradin et al. (Ref. 86) has discovered a connection of H3K27 acetylation in prefrontal neurons with higher hypo-acetylation in OUD brains, leading to silencing of the genes. This process involved 388 loci and was able to distinguish cases from controls with the use of machine-learning classifiers. Despite each case showing unique changes, owing to the convergence analysis five repeatedly impacted genes, namely ASTN2, KCNMA1, DUSP4, GABBR2 and ENOX1 were identified. These findings highlight shared biological pathways (Ref. 86).

Complementary to these findings, animal studies provide evidence that long-term exposure to opioids leads to histone modifications, consistent with modifications seen in human OUD brains (Ref. 82). Collectively, these findings may give a strong implication for the therapeutic potential of histone-modifying drugs. However, the safety and efficacy in use for OUD will have to be investigated extensively.

#### MicroRNA regulation in OUD

MicroRNAs (miRNAs) have also been found as another critical player of epigenetic regulation in OUD. A study conducted among opioid-dependent individuals found that miR-320a and let-7b-5p were significantly upregulated in heroin users (Ref. 87). It has also been found that the let-7 family downregulates OPRM1 translation, which may contribute to opioid tolerance (Ref. 82).

Grimm et al. (Ref. 88), using brain samples, identified 89 dysregulated miRNAs in the prefrontal cortex of individuals with OUD (Ref. 88). These included miR-92a-3p, which targeted many genes related to neuroplasticity (Ref. 87). Mahnke et al. (Ref. 89) have conducted a study in infants, extending the search for miRNA activity. A panel of cord blood miRNAs, such as miR-128-3p and miR-30c-5p, was found to predict the severity of neonatal withdrawal syndrome with high accuracy (AUC up to 0.99) (Ref. 89).

### Therapeutic Implications related to epigenetics

The above findings imply the potential of translational opportunities. Epigenetic modifications such as DNA methylation and miRNA profiles may have potential as biomarkers in predicting the risk, treatment response and even relapse prediction in OUD (Refs 83, 89). Chromatin remodeling underscored the potential of utilizing HDAC inhibitors or mediators aimed at histone-modifying enzymes, although preclinical findings have been consistent (Ref. 86). Therapies based on miRNA such as antagomirs against let-7 might eventually enhance pharmacological treatments such as methadone or buprenorphine. In conclusion, epigenetics provides valuable mechanistic understanding and innovative treatment targets. However, translation of these findings into the clinical setting necessitates additional longitudinal, multi-omic and cross-ancestry studies.

## GWAS studies in the field

Large, global genome-wide association studies (GWAS) have started to identify the highly polygenic architecture of OUD and provide replicable risk loci. When broader meta-analytic datasets were included, cross-ancestry meta-analysis in the Million Veteran Program (MVP) with additional cohorts (such as the Psychiatric Genomics Consortium) revealed genome-wide significant signals at OPRM1 and a novel locus at FURIN, as well as other loci (e.g., RABEPK, FBXW4, NCAM1, KCNN1, TSNARE1) (Ref. 90). Utilizing genetic correlations between OUD, alcohol use disorder and cannabis use disorder, complementary work employing multi-trait GWAS enhanced the power of discovery and produced approximately nineteen independent loci (Ref. 91). When taken as a whole, these efforts put the current total of independent OUD risk loci across single-trait cross-ancestry and multi-trait frameworks at about 14–19, which is an order of magnitude higher than previous research (Refs 90, 91).

OPRM1 rs1799971 (A118G/Asn40Asp), a long-standing candidate variant, now has strong support because it was recaptured in subsequent meta-analyses (Refs 90, 91) and achieved genome-wide significance in MVP European-ancestry samples with replication in two independent cohorts (Ref. 92). The importance of very large samples and strict control for population structure is highlighted by the fact that, significantly, genome-wide signals still concentrate at OPRM1 (and FURIN) across the largest analyses to date, while a number of previously discussed candidates have not achieved genome-wide significance for OUD in single-trait analyses (Refs 90, 91).

These findings support the idea that OUD is extremely polygenic, meaning that meaningful prediction necessitates combining data from the entire genome, and that individual common variants have negligible effects. In line with an early-stage predictor for a complex, multifactorial trait, polygenic risk scores (PRS) derived from current OUD GWAS explain a modest fraction of variance (2.4–3.8% in case status across European-ancestry test sets) (Refs 93, 94). PRS performance should increase as discovery GWAS expands and diversifies; however, OUD PRS currently offers little additional predictive value beyond conventional clinical and environmental factors and is not clinically actionable (Ref. 93).

Due to allele frequency and Linkage Disequilibrium (LD) differences, PRS accuracy is crucially dependent on ancestry match between the discovery and target samples; scores trained primarily in European cohorts perform worse in other populations, underscoring the urgent need for ancestrally diverse OUD GWAS (Refs 95, 96). It is apparent that GWAS consistently demonstrates genetic associations between OUD and other substance use and mental health conditions, indicating a shared polygenic liability that may eventually inform individualised, integrated prevention and treatment strategies (Refs 90, 97).

## Limitations related to available studies

According to Vereczkei et al. (Ref. 61), Hou & Li (Ref. 37), Levran et al. (Ref. 44), Zhang et al. (Ref. 45) and Beer et al. (Ref. 43), several early candidate–gene reports (including studies of OPRD1 and DRD2) used cohorts of approximately 300–600 cases (with comparable controls). These studies were underpowered, which helps explain inconsistent replication across cohorts and ancestries because OUD risk variants exert small effects (OR ≈ 1.05–1.15). This picture has been clarified by recent large-scale GWAS (PGC/MVP and others), which have confirmed signals such as OPRM1 while finding modest support for a number of previously suggested candidate loci when tested at sufficient scale with strict stratification control (Refs 90, 91, 92, 97).

A persistent problem in OUD genetics has been the lack of replication: in the largest GWAS/meta-analyses to date, genome-wide significant signals concentrate at OPRM1 (and FURIN), while previously suggested candidates like OPRD1 and DRD2/ANKK1 failed to achieve genome-wide significance for OUD in single-trait analyses (Refs 90, 91, 92). For instance, OPRD1 and DRD2/ANKK1 were not included in the genome-wide significant set of a cross-ancestry Million Veteran Program meta-analysis (N ≈ 425,944), which revealed genome-wide loci at OPRM1, FURIN, RABEPK, FBXW4, NCAM1, KCNN1 and TSNARE1 (Ref. 90). OPRM1 and FURIN were also found to be the main single-variant signals in a complementary meta-analysis (total N ≈ 639,063; 20,686 OUD cases) (Ref. 91).

This calls into question the accuracy of those early reports and emphasises the necessity of interpreting them with caution (Refs 98, 99, 100, 101). In the past, false-positive associations could have been caused by potential confounding factors such as population stratification (differences in ancestry between cases and controls) (Refs 102, 103, 104). Future research must address this by emphasizing independent replication, pre-registering analyses when feasible and rigorously controlling population structure and other covariates (Refs 102, 105, 106). The significance of large, ancestrally diverse GWAS and replication is further supported by the fact that, despite the large number of candidate–gene associations that have been proposed for OUD, many of them suffer from small sample sizes, lack of replication across cohorts and potential stratification biases (Refs 101, 107).

These insights further suggest that while there is significant progress in this field of study, there is a requirement for more robust evidence.

## Conclusion

Current studies indicate that genetics plays a significant role in the development of opioid addiction, but social, psychological and environmental factors should not be overlooked. Numerous research indicates that the OPRM1, OPRD1, OPRK1, PDYN, OPRL1 and POMC genes of the opioidergic system, the DRD1, DRD2, DRD3, DRD4, ANKK1 and COMT genes of the dopaminergic system, and the GABRA2, GABRB3, GABRG2, GAD1 and GAD2 genes of the GABAergic system are associated with the phenotypes of OUD in various ethnic groups. In addition, the genetic mechanism that is influenced by the environment, i.e., epigenetics, is gaining prominence in genetic studies of addiction. Further, the evidence suggests that chronic opioid use is associated with epigenetic changes in the DNA of brain tissues’ post-translational histone modifications and DNA methylation processes. The dopamine-mesolimbic motivation–reward–reinforcement cycle remains the most consistent physiological theory in addiction, with the opioidergic, dopaminergic and GABAergic systems driving this mechanism. Each class and subclass of addictive substances has its own pharmacokinetics, psychoactive properties and role within the dopamine–mesolimbic system.

Moreover, these data demonstrate that strong, repeatable OUD genetics findings have mostly come from suitably powered, ancestrally diverse GWAS that consistently implicate OPRM1 (and FURIN), even though many historical candidate-gene signals (e.g., OPRD1, DRD2/ANKK1) lack genome-wide support. In the future, rigorous population-structure control, replication across ancestries and pre-registered analyses will be essential to converting statistical associations into mechanistic understanding and clinical utility. The development of precisely targeted drugs and psychological techniques to address the worldwide opioid addiction epidemic will be made possible by an understanding of the genetics, epigenetics and neurobiology of addiction.

There are still a lot of unanswered questions despite these advancements. It is still unclear how many risk variants contribute to OUD, particularly when they interact with environmental exposures like chronic stress or early-life trauma. Further research is needed to understand how environmental triggers alter the epigenome to affect the onset, progression and risk of relapse of OUD, as well as the interaction between genetic susceptibility and epigenetic modifications. In order to capture dynamic gene environment, epigenome interactions over time and guarantee that findings are globally applicable, future research should prioritise longitudinal, multi-omics and multi-ancestry approaches.

Another unresolved issue is translational applications. Currently, treatment selection is rarely guided by genetic and epigenetic findings. Utilizing genetic profiles or epigenetic markers to forecast treatment response is still a promising but unexplored goal in personalised addiction medicine. Developing epigenetic interventions to reverse maladaptive transcriptional states in the brain’s reward circuits or customizing pharmacotherapy (such as methadone, buprenorphine or naltrexone) based on genotype are two approaches. Coordinated international research, data exchange and replication across various populations will be necessary for such therapeutic advancements.

In conclusion, the field needs to move toward incorporating these discoveries into a mechanistic and clinically applicable framework, even though noteworthy progress has been made in identifying the genetic and epigenetic architecture of OUD. To fully utilise genetic and epigenetic research in the fight against opioid addiction, it will be crucial to close the gap between association studies and practical treatment.

## Supporting information

Ranadeva et al. supplementary material Ranadeva et al. supplementary material

## Supplementary material

The supplementary material for this article can be found at http://doi.org/10.1017/erm.2025.10024.
