# Supplementary material for: Genetic and Epigenetic Approaches to Opioid Use Disorder
Source: Expert Rev Mol Med. 2025 Sep 25;27:e35. doi: 10.1017/erm.2025.10024 (PMC12571026; doi:10.1017/erm.2025.10024)
Supplement: Ranadeva et al. supplementary material [file S1462399425100240sup001.zip › Supplementary Table S1,..docx]

Table I Genes and SNVs of the reward system of OUD which have demonstrated an association.

| **System** | **Gene** | **Function** | **SNVs** | **Association** | **References** |
| --- | --- | --- | --- | --- | --- |
| Opioidergic | OPRMI | Receptor | rs1799972 | Predominantly associated in individuals of African descent (p<0.006) and observed increased frequency of opioid addiction among multiple groups of African American, Caucasian, Hispanic, Native North American or Other ethnicities. | [1,2] |
|  |  |  | rs1799971 | Well studied variant among varying ethnic groups and more common in non-African populations. | [3] [4] [5,6] |
|  |  |  | rs3778150 | Significantly associated with opioid dependence in a African-Americans and European-Americans and with heroin dependence among multiple groups; European American, European Australian, and African American participants (p= 4.3e-8). | [2] |
|  | OPRKI | Receptor | G36T | Two haplotypes (AGCTCGTC, GGCGTGCC) are significantly associated with opioid addiction in Hispanics. The variant is slightly higher in opioid-dependent Caucasians and Hispanics. | [2] |
|  | PDYN | Ligand | rs1022563 | Significantly associated with opioid addiction in European-Americans. | [7] |
|  |  |  | rs910080 | Significant associations (ORs) for rs910080 and rs199774 were found in female opioid addicts as well as European-Americans (p<0.05). | [7] |
|  |  |  | rs199774 |  |  |
|  | OPRD1 | Receptor | rs2236861 | Highly associated with opioid abuse or dependence risk among European population (p=0.001) and heroin dependence among multiple groups, US Caucasians and Israeli Jewish cases with US Caucasian controls. (p= 0.002). | [8,9] |
|  |  |  | rs2236857 | Significantly associated with opioid dependence. | [10] |
|  |  |  | rs3766951 | Significantly associated with opioid dependence. | [10] |
|  |  |  | rs2234918 | The rs2234918 variant is rarely associated with dependence in the German and European populations (p<0.001) | [11,12] |
|  |  |  | rs1042114 | rs1042114 is associated with opioid dependence in European-Americans. The GCAACT haplotype, which consists of both rs1042114 and rs2234918, is more prevalent in cases of opioid dependence than in controls. | [11] |
|  | OPRL1 | Receptor | rs6090041 | Significantly associated with European population (p= 0.03) for opioid exposure and opiate addiction among Scandinavian population. | [13] |
|  |  |  | rs6090043 | Significantly associated pointwise with opiate addiction among Scandinavian population | [14] |
|  | POMC | Protein | rs934778 | Specifically associated with opiate dependence and associated with minor allele frequencies of 0.30 and 0.27. | [15] |
|  |  |  | rs10009388 |  |  |
| GABA-ergic | GABRA2 | Receptor | rs11503014 | Associated heroin addiction in African American (p= 0.001) | [16] |
|  | GABRB3 | Receptor | rs4906902,  rs8179184, rs20317 | Contribute to the pathogenesis of Heroin dependence | [16] |
|  |  |  | rs7165224 | Associated with heroin addiction among African American population (p=0.01). | [17] |
|  | GABRG2 | Receptor | rs211014 | Associated with heroin dependence in a American (P=0.0005) and Chinese male population (P = 0.015). | [18,19] |
|  | GAD1 | Protein (Glutamic acid decarboxylase1) | rs1978340, rs3762556, rs3791878, rs3749034,  rs2241165 | Associated with development of heroin dependence was reported in the Han Chinese sample. | [20] |
|  |  |  | rs2058725 | Associated with heroin addiction among African Americans (p=0.0074) | [17] |
|  | GAD2 | Protein (Glutamic acid decarboxylase 2) | rs8190646 | Associated with heroin addiction among African Americans (p=0.0066) | [17] |
| Dopaminergic | COMT | Enzyme | rs4680 | Significant association of the G/A and A/A genotypes with opiate addiction in women, but not in men, among Hispanic subjects with opiate dependence. This result confirmed a previous association report for this variant in an Israeli population (p0.05). Associated with opioid addiction among European population (p= 0.0028) | [21,22] |
|  | DRD1 | Receptor | rs5326 | Associated with heroin addiction in African Americans and there is association of rapidity of the heroin dependence with the first dose use among Han Chinese population | [17] [23] |
|  |  |  | rs686 | Associated mixed population of Caucasians and African Americans (p<0.05) | [23] |
|  | DRD2 | Receptor | rs1079597 | rs1800497 and rs1079597 are inherited together more often. Both variants have been associated with opioid dependence in Han Chinese.  Associated with European and Chinese (p<0.05) | [24–28] |
|  |  |  | rs1800497 | Associated with opioid addiction and it is a risk factor for OUD among Chinese, European (p<0.05). | [28–30] |
|  | DRD3 | Receptor |  | Associated with opioid addiction in terms of increased drug seeking which is a risk factor. | [31] |
|  | DRD4 | Receptor |  | The 7-repeat variants of DRD4 exon III long repeat alleles are more prevalent in opioid-dependent populations. | [31] |
|  |  |  | rs1800955 | Found to be nominally associated with opioids among Hungarian patients. | [28] |
|  | ANKK1 | Protein | rs1800497 | rs1800497 and rs1079597 are inherited together more often. Both variants have been associated with opioid dependence in Han Chinese. | [24,25] |

**References**

[1] Bond C, Laforge KS, Tian M, Melia D, Zhang S, Borg L, et al. Single-nucleotide polymorphism in the human mu opioid receptor gene alters β-endorphin binding and activity: Possible implications for opiate addiction. Proc Natl Acad Sci U S A 1998;95:9608–13. https://doi.org/10.1073/pnas.95.16.9608.

[2] Crystal HA, Hamon S, Randesi M, Cook J, Anastos K, Lazar J, et al. A C17T polymorphism in the mu opiate receptor is associated with quantitative measures of drug use in African American women. Addiction Biology 2012;17:181–91. https://doi.org/10.1111/j.1369-1600.2010.00265.x.

[3] Kumar D, Chakraborty J, Das S. Epistatic effects between variants of kappa-opioid receptor gene and A118G of mu-opioid receptor gene increase susceptibility to addiction in Indian population. Prog Neuropsychopharmacol Biol Psychiatry 2012;36:225–30. https://doi.org/10.1016/J.PNPBP.2011.10.018.

[4] Bond C, Laforge KS, Tian M, Melia D, Zhang S, Borg L, et al. Single-nucleotide polymorphism in the human mu opioid receptor gene alters beta-endorphin binding and activity: possible implications for opiate addiction. Proc Natl Acad Sci U S A 1998;95:9608–13. https://doi.org/10.1073/PNAS.95.16.9608.

[5] Nagaya D, Ramanathan S, Ravichandran M, Navaratnam V. A118G mu opioid receptor polymorphism among drug addicts in Malaysia. Http://DxDoiOrg/101142/S0219635212500082 2012;11:117–22. https://doi.org/10.1142/S0219635212500082.

[6] Clarke TK, Crist RC, Kampman KM, Dackis CA, Pettinati HM, O’Brien CP, et al. Low frequency genetic variants in the μ-opioid receptor (OPRM1) affect risk for addiction to heroin and cocaine. Neurosci Lett 2013;542:71–5. https://doi.org/10.1016/J.NEULET.2013.02.018.

[7] Clarke TK, Ambrose-Lanci L, Ferraro TN, Berrettini WH, Kampman KM, Dackis CA, et al. Genetic association analyses of PDYN polymorphisms with heroin and cocaine addiction. Genes Brain Behav 2012;11:415–23. https://doi.org/10.1111/J.1601-183X.2012.00785.X.

[8] Beer B, Erb R, Pavlic M, Ulmer H, Giacomuzzi S, Riemer Y, et al. Association of Polymorphisms in Pharmacogenetic Candidate Genes (OPRD1, GAL, ABCB1, OPRM1) with Opioid Dependence in European Population: A Case-Control Study. PLoS One 2013;8. https://doi.org/10.1371/journal.pone.0075359.

[9] Levran O, Londono D, O’Hara K, Nielsen DA, Peles E, Rotrosen J, et al. Genetic susceptibility to heroin addiction: A candidate gene association study. Genes Brain Behav 2008;7:720–9. https://doi.org/10.1111/j.1601-183X.2008.00410.x.

[10] Reed B, Butelman ER, Yuferov V, Randesi M, Kreek MJ. Genetics of Opiate Addiction. Curr Psychiatry Rep 2014;16. https://doi.org/10.1007/S11920-014-0504-6.

[11] Zhang H, Kranzler HR, Yang BZ, Luo X, Gelernter J. The OPRD1 and OPRK1 loci in alcohol or drug dependence: OPRD1 variation modulates substance dependence risk. Mol Psychiatry 2008;13:531–43. https://doi.org/10.1038/SJ.MP.4002035.

[12] Mistry CJ, Bawor M, Desai D, Marsh DC, Samaan Z. Genetics of Opioid Dependence: A Review of the Genetic Contribution to Opioid Dependence. Curr Psychiatry Rev 2014;10:156. https://doi.org/10.2174/1573400510666140320000928.

[13] Briant JA, Nielsen DA, Proudnikov D, Londono D, Ho A, Ott J, et al. Evidence for association of two variants of the nociceptin/orphanin FQ receptor gene OPRL1 with vulnerability to develop opiate addiction in Caucasians. Psychiatr Genet 2010;20:65–72. https://doi.org/10.1097/YPG.0b013e32833511f6.

[14] Briant JA, Nielsen DA, Proudnikov D, Londono D, Ho A, Ott J, et al. Evidence for association of two variants of the nociceptin/orphanin FQ receptor gene OPRL1 with vulnerability to develop opiate addiction in Caucasians. Psychiatr Genet 2010;20:65–72. https://doi.org/10.1097/YPG.0B013E32833511F6.

[15] Xuei X, Flury-Wetherill L, Bierut L, Dick D, Nurnberger J, Foroud T, et al. The opioid system in alcohol and drug dependence: Family-based association study. American Journal of Medical Genetics Part B: Neuropsychiatric Genetics 2007;144B:877–84. https://doi.org/10.1002/AJMG.B.30531.

[16] Smelson D, Yu L, Buyske S, Gonzalez G, Tischfield J, Deutsch CK, et al. Genetic association of GABA-A receptor alpha-2 and Mu opioid receptor with cocaine Cue-reactivity: Evidence for inhibitory synaptic neurotransmission involvement in cocaine dependence. American Journal on Addictions 2012;21:411–5. https://doi.org/10.1111/j.1521-0391.2012.00253.x.

[17] Levran O, Londono D, O’Hara K, Randesi M, Rotrosen J, Casadonte P, et al. Heroin addiction in African Americans: a hypothesis-driven association study. Genes Brain Behav 2009;8:531. https://doi.org/10.1111/J.1601-183X.2009.00501.X.

[18] Li D, Sulovari A, Cheng C, Zhao H, Kranzler HR, Gelernter J. Association of Gamma-Aminobutyric Acid A Receptor α2 Gene (GABRA2) with Alcohol Use Disorder. Neuropsychopharmacology 2014;39:907. https://doi.org/10.1038/NPP.2013.291.

[19] Loh EW, Tang NLS, Lee DTS, Liu SI, Stadlin A. Association analysis of GABA receptor subunit genes on 5q33 with heroin dependence in a Chinese male population. Am J Med Genet B Neuropsychiatr Genet 2007;144B:439–43. https://doi.org/10.1002/AJMG.B.30429.

[20] Wu W, Zhu YS, Li SB. Polymorphisms in the glutamate decarboxylase 1 gene associated with heroin dependence. Biochem Biophys Res Commun 2012;422:91–6. https://doi.org/10.1016/J.BBRC.2012.04.112.

[21] Christoffersen DJ, Damkier P, Feddersen S, Möller S, Thomsen JL, Brasch-Andersen C, et al. The ABCB1, rs9282564, AG and TT Genotypes and the COMT, rs4680, AA Genotype are Less Frequent in Deceased Patients with Opioid Addiction than in Living Patients with Opioid Addiction. Basic Clin Pharmacol Toxicol 2016;119:381–8. https://doi.org/10.1111/bcpt.12602.

[22] Oosterhuis BE, LaForge KS, Proudnikov D, Ho A, Nielsen DA, Gianotti R, et al. Catechol-O-Methyltransferase (COMT) Gene Variants: Possible Association of the Val158Met Variant With Opiate Addiction in Hispanic Women. Am J Med Genet B Neuropsychiatr Genet 2008;147B:793. https://doi.org/10.1002/AJMG.B.30716.

[23] Jacobs MM, Ökvist A, Horvath M, Keller E, Bannon MJ, Morgello S, et al. Dopamine receptor D1 and postsynaptic density gene variants associate with opiate abuse and striatal expression levels. Mol Psychiatry 2013;18:1205–10. https://doi.org/10.1038/MP.2012.140.

[24] Zhang J, Yan P, Li Y, Cai X, Yang Z, Miao X, et al. A 35.8 kilobases haplotype spanning ANKK1 and DRD2 is associated with heroin dependence in Han Chinese males. Brain Res 2018;1688:54–64. https://doi.org/10.1016/J.BRAINRES.2018.03.017.

[25] Tsou CC, Chou HW, Ho PS, Kuo SC, Chen CY, Huang CC, et al. DRD2 and ANKK1 genes associate with late-onset heroin dependence in men. World J Biol Psychiatry 2019;20:605–15. https://doi.org/10.1080/15622975.2017.1372630.

[26] Xu K, Lichtermann D, Lipsky RH, Franke P, Liu X, Hu Y, et al. Association of specific haplotypes of D 2 dopamine receptor gene with vulnerability to heroin dependence in 2 distinct populations. Arch Gen Psychiatry 2004;61:597–606. https://doi.org/10.1001/ARCHPSYC.61.6.597.

[27] Wang N, Zhang JB, Zhao J, Cai XT, Zhu YS, Li SB. Association between dopamine D2 receptor gene polymorphisms and the risk of heroin dependence. Genetics and Molecular Research 2016;15. https://doi.org/10.4238/GMR15048772.

[28] Vereczkei A, Demetrovics Z, Szekely A, Sarkozy P, Antal P, Szilagyi A, et al. Multivariate Analysis of Dopaminergic Gene Variants as Risk Factors of Heroin Dependence. PLoS One 2013;8. https://doi.org/10.1371/JOURNAL.PONE.0066592.

[29] Teh LK, Izuddin AF, Fazleen HMH, Zakaria ZA, Salleh MZ. Tridimensional personalities and polymorphism of dopamine D2 receptor among heroin addicts. Biol Res Nurs 2012;14:188–96. https://doi.org/10.1177/1099800411405030.

[30] Doehring A, Hentig N Von, Graff J, Salamat S, Schmidt M, Geisslinger G, et al. Genetic variants altering dopamine D2 receptor expression or function modulate the risk of opiate addiction and the dosage requirements of methadone substitution. Pharmacogenet Genomics 2009;19:407–14. https://doi.org/10.1097/FPC.0B013E328320A3FD.

[31] Duaux E, Gorwood P, Griffon N, Bourdel MC, Sautel F, Sokoloff P, et al. Homozygosity at the dopamine D3 receptor gene is associated with opiate dependence. Mol Psychiatry 1998;3:333–6. https://doi.org/10.1038/SJ.MP.4000409.
