# Supplementary figures and images for: Genetic and Epigenetic Approaches to Opioid Use Disorder
Source: Expert Rev Mol Med. 2025 Sep 25;27:e35. doi: 10.1017/erm.2025.10024 (PMC12571026; doi:10.1017/erm.2025.10024)

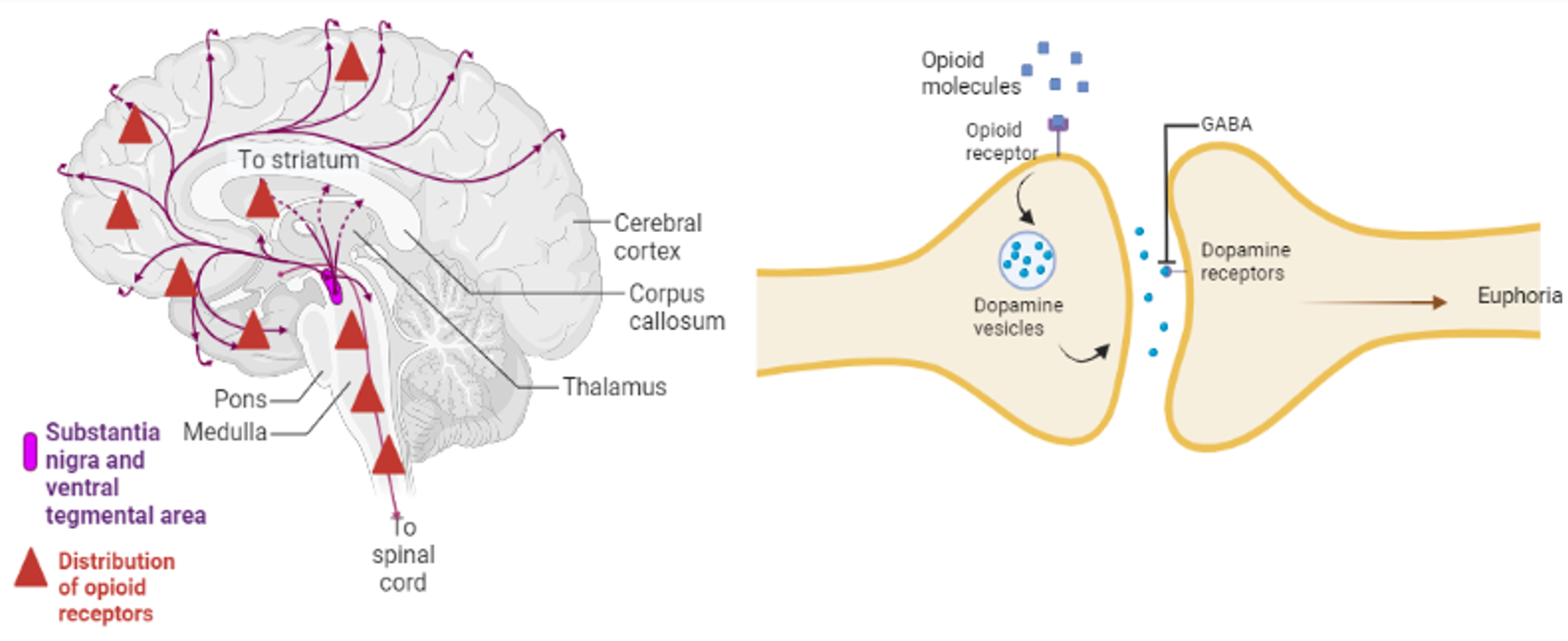

Supplement: Ranadeva et al. supplementary material [file S1462399425100240sup001.zip › Supplementary Figure S1, .png]
